# Anthrax Lethal Factor Cleaves Mouse Nlrp1b in Both Toxin-Sensitive and Toxin-Resistant Macrophages

**DOI:** 10.1371/journal.pone.0049741

**Published:** 2012-11-12

**Authors:** Kristina A. Hellmich, Jonathan L. Levinsohn, Rasem Fattah, Zachary L. Newman, Nolan Maier, Inka Sastalla, Shihui Liu, Stephen H. Leppla, Mahtab Moayeri

**Affiliations:** 1 Microbial Pathogenesis Section, Laboratory of Parasitic Diseases, National Institute of Allergy and Infectious Diseases, National Institutes of Health, Bethesda, Maryland, United States of America; Wadsworth Center, New York State Dept. Health, United States of America

## Abstract

Anthrax lethal factor (LF) is the protease component of anthrax lethal toxin (LT). LT induces pyroptosis in macrophages of certain inbred mouse and rat strains, while macrophages from other inbred strains are resistant to the toxin. In rats, the sensitivity of macrophages to toxin-induced cell death is determined by the presence of an LF cleavage sequence in the inflammasome sensor Nlrp1. LF cleaves rat Nlrp1 of toxin-sensitive macrophages, activating caspase-1 and inducing cell death. Toxin-resistant macrophages, however, express Nlrp1 proteins which do not harbor the LF cleavage site. We report here that mouse Nlrp1b proteins are also cleaved by LF. In contrast to the situation in rats, sensitivity and resistance of Balb/cJ and NOD/LtJ macrophages does not correlate to the susceptibility of their Nlrp1b proteins to cleavage by LF, as both proteins are cleaved. Two LF cleavage sites, at residues 38 and 44, were identified in mouse Nlrp1b. Our results suggest that the resistance of NOD/LtJ macrophages to LT, and the inability of the Nlrp1b protein expressed in these cells to be activated by the toxin are likely due to polymorphisms other than those at the LF cleavage sites.

## Introduction

Anthrax lethal toxin (LT) consists of a receptor-binding protein, protective antigen (PA), and a protease, lethal factor (LF). LT is a major virulence factor, and its injection alone is sufficient to induce the vascular collapse associated with anthrax disease in animal models (for review see [1]). Rodent macrophages from certain inbred strains are rapidly lysed by LT. Susceptibility to LT is controlled by alleles encoding variants of the NOD-like receptor (NLR) Nlrp1b in mice [2], and its ortholog in rats [3]. Nlrp proteins are intracellular pattern recognition receptors that detect a variety of signals associated with pathogens and other dangers to the cell [4]. While a wide range of stimuli are recognized by intracellular sensors such as Nlrp3 [4], the only known activator of rodent Nlrp1 is LT. Treatment of macrophages and dendritic cells from some inbred rodent strains such as Balb/cJ (mice) and Fischer (rats) to LT results in Nlrp1/Nlrp1b-mediated activation of caspase-1 and subsequent cleavage of IL-1β and IL-18, initiating an immune response concurrent with rapid cell death (pyroptosis) [2], [5]. Macrophages from other rodent strains such as C57BL/6J and NOD/LtJ (mice) and Lewis (rats) express Nlrp1 variants which are not activated by LT and these cells are resistant to the toxin [2], [5]. Our laboratory recently demonstrated that LT sensitivity in rats is determined by polymorphisms at residues 40-48 in the N-terminus of rat Nlrp1 [6]. LF cleaves this site in rat Nlrp1 of inbred strains harboring LT-sensitive macrophages, but not the Nlrp1 of LT-resistant strains [6].

Mice harbor three Nlrp1 paralogs in their genomes, but the existing data indicate that only *Nlrp1b* controls LT sensitivity [2]. Mouse Nlrp1b proteins are highly polymorphic [2], unlike the situation in rats, where Nlrp1 is almost identical in all strains except in the small region in the N-terminus noted above. Sequencing *Nlrp1b* from 18 inbred mouse strains identified five different Nlrp1b protein sequences, two associated with LT sensitivity and three with resistance [2]. The proteins encoded by four of the five different *Nlrp1b* alleles have >88% homology to one another. Especially intriguing is the high degree of homology between proteins encoded by *Nlrp1b* allele 1 (expressed in Balb/cJ and numerous other strains with LT-sensitive macrophages), *Nlrp1b* allele 3 (expressed in NOD/LtJ and AKR/J mice, which harbor LT-resistant macrophages) and *Nlrp1b* allele 5 (expressed in CAST/EiJ mice, which harbor LT-sensitive macrophages). We wished to determine whether the differential responses of these highly homologous proteins were determined by LF cleavage of Nlrp1b, as it is in rats [6]. We report here that Nlrp1b expressed in both Balb/cJ macrophages (LT-sensitive) as well as Nlrp1b from NOD/LtJ macrophages (LT-resistant) are cleaved by LT within the N-terminal region, at the same sites. These results suggest that the resistance of NOD/LtJ macrophages to LT, and the inability of the Nlrp1b protein expressed in these cells to be activated by the toxin, is likely due to polymorphisms that render the protein inactive in a manner independent of LT cleavage.

**Figure 1:**
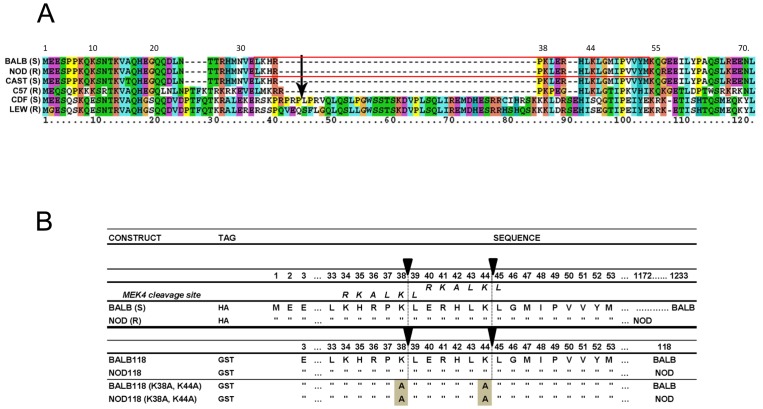
Nlrp1 protein alignments and constructs. (A). Alignment of amino acid sequences from the N-terminus of mouse Nlrp1b and rat Nlrp1 proteins. Sequences shown are those of 4 mouse and 2 rat strains, including strains having macrophages that are either sensitive (S) or resistant (R) to LT. The previously identified LT cleavage site after residue 44 in rat CDF Nlrp1 is indicated by an arrow. The red box indicates the region of mouse sequence shown in (B). (B) Nlrp1b constructs used in this study with focus on N-terminal regions containing putative LF cleavage sites. The top two constructs represent the full-length HA-tagged Nlrp1b proteins from the LT-sensitive Balb/cJ (BALB) and the LT-resistant NOD/LtJ (NOD) macrophages, which were expressed in HT1080 cells. Full length NOD Nlrp1b is shorter (1172 aa) than BALB Nlrp1b due to a region downstream of the leucine rich repeat domain that is missing in this protein. The next four constructs represent proteins where aa 3-118 of Nlrp1b were expressed and purified from *E. coli* as N-terminal GST-tagged proteins. These proteins also contain a C-terminal His6 tag (not represented in figure). In the sequence alignments, residues identical to those in the construct listed above are indicated by quotation marks (“). Putative LF cleavage sites based on previously described motifs are drawn as vertical dotted lines below filled arrows. The MEK4 cleavage site is also aligned with both putative Nlrp1b cleavage sites. The last two sequences are those of constructs having two key lysine residues substituted with alanine.

## Results and Discussion

**Figure 2:**
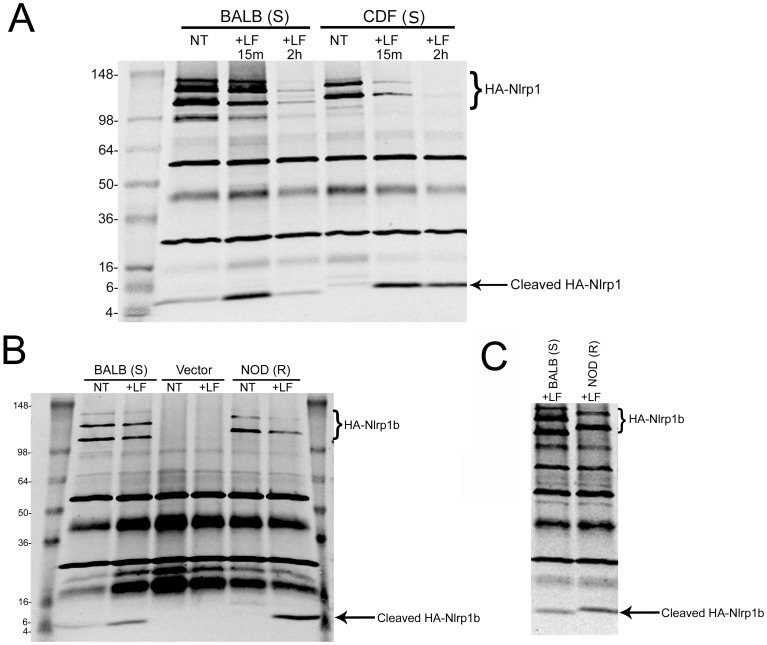
Cleavage of full length rat and mouse Nlrp1b proteins by LF. (A) IP (anti-HA pulldown) followed by anti-HA Western blotting of lysates from HT1080 cells expressing HA-tagged mouse Nlrp1b (BALB) or rat Nlrp1(CDF) proteins following treatment with LF (1 µg/ml) for 15 min or 2 h. Cleavage of CDF Nlrp1 leads to appearance of a 6-kDa HA-reactive band and cleavage of BALB Nlrp1b leads to a slightly smaller fragment. (B) IP (anti-HA pulldown) followed by anti-HA Western blotting of lysates from HT1080 cells expressing HA-tagged Nlrp1b proteins or control vector following treatment with LF (1 µg/ml, 30 min). Anti-HA cross-reactive bands not marked as HA-Nlrp1 also appear in vector-transfected controls. (C) Comparison of size of cleavage fragments generated after cleavage of BALB and NOD HA-tagged Nlrp1b (using conditions same as 2B), indicating the smaller size of the fragment generated following cleavage of the BALB protein (Western representative of five similar experiments).

Our earlier studies identified an LF cleavage site within the N-terminus of rat Nlrp1. Toxin cleavage at this site leads to macrophage pyroptosis [6]. An alignment of rat Nlrp1 sequences of Fischer (CDF, LT-sensitive) and Lewis (LEW, LT-resistant) rats, as well as Nlrp1b sequences from four mouse strains is shown in Figure 1A. The alignment shows that the LF cleavage site within rat Nlrp1 lies in an inserted sequence that is absent in mouse Nlrp1b proteins. Perplexingly, unlike the situation in rats, the Balb/cJ (BALB, LT-sensitive) and NOD/LtJ (NOD, LT-resistant) Nlrp1b sequences, present in mice having macrophages of opposing sensitivities to LT, are identical over the first 55 amino acids of the protein. Examination of the BALB (S) and NOD (R) Nlrp1b sequences revealed two nearby potential LF cleavage sites having characteristics like those of the established cleavage sites identified in rat Nlrp1, mitogen activated protein kinase kinase 4 (MEK4), and other LF substrates [6]–[9] (Red box in Figure 1A and Figure 1B). Nlrp1 proteins are expressed endogenously at low levels that are difficult to detect via Western blotting. Therefore we expressed full-length N-terminally hemagglutinin (HA) epitope-tagged rat Nlrp1 (CDF-S) and mouse Nlrp1b (BALB-S and NOD-R) proteins in HT1080 human fibroblasts by stable transfection. Immunoprecipitation (IP) with anti-HA antibodies showed expression of full-length HA-tagged Nlrp1 proteins, as well as multiple C-terminally truncated variants (Figure 2). As previously reported [6], LF cleavage of HA-tagged rat Nlrp1 (CDF) expressed in fibroblasts produced a 6-kDA HA-antibody reactive cleavage fragment (Figure 2A). In a new result, we found that LF cleaved the HA-tagged BALB (S) Nlrp1b protein, producing a (slightly smaller) ∼5-kDa HA-antibody reactive cleavage fragment (Figure 2A), suggesting that the BALB (S) Nlrp1b cleavage site is slightly upstream of the rat Nlrp1 insertion sequence which contains the cleavage site. This would also mean that this cleavage site would lie within a region of identical sequence for both BALB (S) and NOD (R) Nlrp1b.

**Figure 3:**
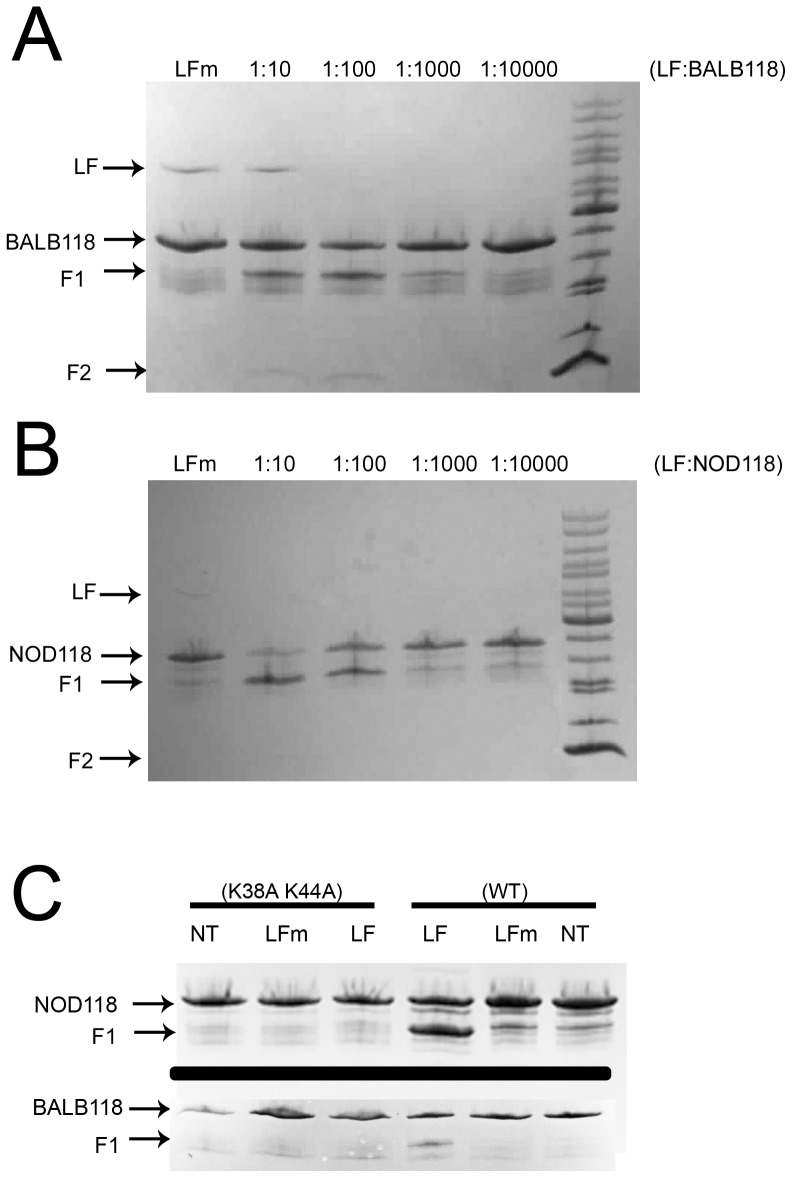
Cleavage of mouse BALB118 and NOD118 Nlrp1 fusion proteins by LF. (A, B) *In vitro* cleavage of N-terminally 6His-GST-tagged aa 3-118 of Nlrp1b proteins. Purified proteins (0.53 mg/ml or 0.94 mg/ml, in A and B, respectively) were treated with the indicated molar ratios of LF, or with a 1∶10 molar ratio of the mutant LF E687C (LFm), for 4 h prior to SDS gel electrophoresis and Coomasie staining. F1 and F2 refer to two fragments generated following LF treatment. (C) GST-tagged or double alanine mutant variants (0.44-0.66 mg/ml) were treated with 33 µg/ml LF or LFm for 4 h prior to SDS gel electrophoresis and Coomassie staining.

We also found that LF cleaves the HA-NOD (R) Nlrp1b protein (Figure 2B), as might be expected given its sequence identity to the HA-BALB (S) protein. Careful assessment in repeated experiments of the fragments generated by LF cleavage of these Nlrp1b proteins showed that the BALB cleavage fragment was slightly smaller than the NOD cleavage fragment, suggesting cleavage at two unique sites within the N-terminal regions of these proteins (Figure 2C). This finding suggested that one or more of the six polymorphisms immediately downstream of the potential LF cleavage sites (with the following amino acid changes: R56K, R67K, L79P, C85Y, I93V, V101I) may influence the site at which LF cleaves these proteins.

**Figure 4:**
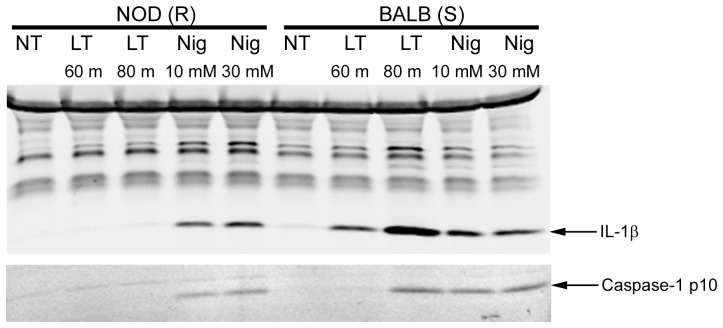
Caspase-1 activation in bone marrow-derived mouse macrophages. LPS-primed (1 µg/ml, 2 h) bone marrow-derived macrophages from Balb/cJ or NOD/LtJ mice were treated with LT (1 µg/ml) for 60 or 80 min, or with nigericin at indicated doses for 20 min. Cell lysates were analyzed by Western blotting for IL-1β, and the same samples were probed with caspase-1 p10 antibody to detect caspase-1 cleavage.

To identify the exact cleavage sites, the first 118 aa of BALB (S) and NOD (R) Nlrp1b were expressed and purified as GST fusions (designated BALB118 and NOD118) (Figure 1B, lines 3 and 4). Both BALB118 and NOD118 were cleaved by LF (Figure 3A and 3B). Interestingly, in repeated experiments we noted that NOD118 appeared to be more efficiently cleaved by LF than BALB118 (data not shown). This result corresponds to a similar phenomenon seen in cell lysates from HT1080 cells expressing full-length HA-Nlrp1b proteins, where NOD (R) Nlrp1b was cleaved more efficiently than BALB (S) Nlrp1b (data not shown). Furthermore, in analyses of the canonical cleavage pathway, where HA-tagged Nlrp1b proteins were immunoprecipitated from cells after delivery of LF to the cytosol by PA, NOD Nlrp1b protein appeared to be more efficiently cleaved (Figure S1).

Mass spectrometry analyses of BALB118 and NOD118 following cleavage by LF yielded masses of 30,486 and 31,257, indicating that LF cleaves between the two lysine-leucine bonds (after K38 and K44) (Figure 1B, arrows). Both cleavage fragments were found following LF treatment of both proteins, albeit with differing prevalence in multiple independent cleavage runs (data not shown). Thus, it appears probable that the two fragments of 5-6 kDa observed following cleavage of the full length Nlrp1b proteins in cell lysates result from cleavage at the K38 and K44 cleavage sites, and that BALB (allele 1) Nlrp1b is preferentially cleaved at the first site while NOD (allele 3) Nlrp1b is preferentially cleaved at the second site. Substitution of the lysines at both sites with alanines (Figure 1B, lines 5, 6) abrogated LF-mediated cleavage of the BALB118 and NOD118 proteins (Figure 3C).

Since the data presented above suggests that Nlrp1b of NOD macrophages would be cleaved by LF delivered to the cytosol, the question arises as to why these cells do not undergo pyroptosis. One possible defect in downstream events could be a failure to activate caspase-1. However, we found that nigericin activated the Nlrp3 inflammasome to elicit efficient activation of caspase-1 and subsequent IL-1β cleavage in both NOD and BALB bone marrow-derived macrophages (Figure 4), indicating that the caspase-1 activation pathway was fully functional. NOD macrophages also had no defect in flagellin-mediated Nlrc4 inflammasome activation (data not shown). As expected, LT activated caspase-1 in BALB (S) but not in NOD (R) macrophages (Figure 4). A possible reason for these findings is that NOD macrophages may be deficient in protein’s that are important to inflammasome assembly in an Nlrp1b-dependent manner, which does not impact Nlrp3-mediated caspase-1 activation.

While this manuscript was in preparation, Frew et al. reported that C-terminal autoproteolysis of Nlrp1b within the FIIND (function-to-find) domain is required for inflammasome activation and that this proteolytic processing was absent in allele 3 (NOD) Nlrp1b due to a single polymorphism, V988D [10]. The site for C-terminal autoproteolysis in Nlrp1 has been identified [11]. Differentially C-terminally truncated variants of NOD (R) Nlrp1b, and BALB (S) Nlrp1b were also present in our expression system (Figure 2). Frew et al. were able to eliminate proteolytic processing of BALB (allele 1) Nlrp1b by introducing the V988D substitution (from NOD, allele 3), and this mutation prevented the protein from being activated by LT [10]. Intriguingly, the authors were unable to restore LF responsiveness to the LT-nonresponsive NOD (R) Nlrp1b protein even after restoration of its autoproteolytic processing. These results, in combination with the findings reported here, suggest that the resistance of NOD (R) Nlrp1b to LF is not due to absence of a required LF cleavage event, or simply due to a deficiency in C-terminal autoproteolysis. It is possible, but unlikely, that preferential LF cleavage of NOD (R) Nlrp1b at residue K44, instead of K38, likely due to the presence of downstream polymorphisms altering folding in the N-terminus of this protein, is the reason for the defect in activation of this protein. It seems more likely that polymorphisms in other domains of this protein render it nonresponsive to LT. The truncated domain downstream of the leucine rich repeats in NOD Nlrp1b may result in altered conformation and folding of this protein in a manner that interferes with its unfolding to allow dimerization or caspase-1 recruitment. Thus, even when autoproteolysis at the C-terminus is restored and LT cleaves the N-terminus efficiently, the protein may be unable to act as an inflammasome platform. The deciphering of the mechanism for resistance to LT requires further experimentation. We propose, however, that cleavage of the N-terminus of both mouse and rat Nlrp1 proteins by LF may be required for activation of the inflammasome by LT, although it may be insufficient in the absence of other processing events.

## Materials and Methods

### Ethics Statement

This study was carried out in strict accordance with the recommendations in the Guide for the Care and Use of Laboratory Animals of the National Institutes of Health. All bone marrow harvests were performed in accordance to protocols approved by the NIAID Animal Care and Use Committee.

### Materials

PA, LF, and LF E687C purification from avirulent *Bacillus anthracis* strains has been described [12]. Concentrations of LT correspond to the concentration of each toxin component (i.e., 1 µg/ml LT has 1 µg/ml PA and 1 µg/ml LF). GST-fusion proteins of BALB118 and NOD118 (described below) were expressed from pGEX-KG vectors in *Escherichia coli* BL21(DE3) and purified in a two-step process on glutathione-Sepharose and nickel chelate columns using standard purification protocols. High affinity anti-HA (cat# 11867423001, Roche Diagnostics, Indianapolis, IN), anti-IL-1β (cat# AF-401-NA, R&D Systems, Minneapolis, MN) and various IR-dye conjugated secondary antibodies (Licor Biosciences, Lincoln, NE and Rockland Immunochemicals, Gilbertsville, PA) were purchased. Nigericin was purchased from Calbiochem (San Diego, CA).

### Cell Culture and Transfections

Cell culture and transfection methods have been previously described [6]. For stable transfections, Nlrp1b-expressing cell lines were derived by selection with hygromycin B (500 µg/ml; Invitrogen) for 15 days. Western blot with anti-HA antibody was performed to identify expression levels. Bone marrow-derived macrophages (BMDMs) were generated from marrow obtained from Balb/cJ or Nod/LtJ mice (Jackson Laboratories, Bar Harbor, ME) as previously described [13].

### Constructs

cDNA sequences for BALB and NOD mNlrp1b were synthesized by GeneArt Life Technologies (Grand Island, NY) and were cloned into the pIREShyg3 vector using Nhe1 and Xma1 sites. BALB118 and NOD118 sequences were synthesized by GeneArt Life Technologies and cloned along with added C-terminal His6 tags into the pGEX-KG vector using BamHI and EcoRI sites. Mutagenesis was performed using the QuikChange system (Agilent Technologies, La Jolla, CA) and sequencing was performed by Macrogen (Rockville, MD).

### Cleavage Assays

To assess Nlrp1 cleavage in cell lysates, cells were grown to confluence and lysed in sucrose buffer (250 mM sucrose, 10 mM HEPES, 0.05 M EDTA, 0.2% Nonidet-P40) containing ZnCl_2_ (1 µM) and NaCl (5 mM), followed by LF treatment at 37°C for varying times. Alternatively, cells were first treated with LT at 1 µg/ml for 5 h (canonical cleavage), followed by lysis in sucrose buffer containing 5 ng/ml LF inhibitor PT-168541-1 (gift of Alan Johnson, Panthera Biopharma). Cleavage reactions were analyzed by Western blot (WB) or immunoprecipitatoin (IP) followed by WB. For *in vitro* cleavage assays with purified proteins, BALB118 and NOD118 were incubated for varying times at 37°C with purified LF at varying concentrations in the presence of ZnCl_2_ (1 µM) and NaCl (5 mM). Samples were separated on an 8-25% SDS-PAGE gel using the PhastSystem (GE Life Sciences, Piscataway, NJ) and visualized by Coomassie staining.

### Western Blots and Immunoprecipitation

WB were performed using either anti-HA (1∶1000), anti-caspase-1 (1∶200), or anti-IL-1β (1∶2,500) and proteins were detected using the Odyssey Infrared Imaging System (Licor Biosciences). For IP, anti-HA antibody (Roche Diagnostics) was added to cell lysates (5-15 µg/ml) and samples were continuously mixed by rotation at 4°C for 1 h, followed by Protein A/G agarose (Santa Cruz Biotechnology) addition and continued overnight 4°C incubation with rotation. Beads were centrifuged at 4,000 rpm for 2 min and washed with 10 mM HEPES three times prior to elution of proteins using SDS loading buffer (10% SDS, 0.6 M DTT, 30% glycerol, 0.012% bromophenol blue, at 90°C, 5 min).

### Mass Spectrometry

The molecular masses of the BALB118 and NOD118 proteins and their cleavage products were determined by liquid chromatography-electrospray mass spectrometry using an HP/Agilent 1100 MSD instrument (Hewlett Packard, Palo Alto, CA) at the NIDDK core facility, Bethesda, MD.

## Supporting Information

Figure S1 **Canonical cleavage of full length mouse Nlrp1b proteins by LT. HT1080 cells expressing HA-tagged mouse Nlrp1b (BALB or NOD) proteins were first treated with LF+PA (1 µg/ml, each) for 3 h.** IP (anti-HA pulldown) was then performed on lysates followed by anti-HA Western blotting.
(TIF)

## References

[1] Moayeri M, Leppla SH (2009) Cellular and systemic effects of anthrax lethal toxin and edema toxin. Mol Aspects Med 30: 439–455. 19638283 10.1016/j.mam.2009.07.003 PMC2784088

[2] Boyden ED, Dietrich WF (2006) Nalp1b controls mouse macrophage susceptibility to anthrax lethal toxin. Nat Genet 38: 240–244. 16429160 10.1038/ng1724

[3] Newman ZL, Printz MP, Liu S, Crown D, Breen L, et al (2010) Susceptibility to anthrax lethal toxin-induced rat death is controlled by a single chromosome 10 locus that includes *rNlrp1* . PLoS Pathog 6: e1000906. 20502689 10.1371/journal.ppat.1000906 PMC2873920

[4] Horvath GL, Schrum JE, De Nardo CM, Latz E (2011) Intracellular sensing of microbes and danger signals by the inflammasomes. Immunol Rev 243: 119–135. 21884172 10.1111/j.1600-065X.2011.01050.x PMC3893570

[5] Newman ZL, Crown D, Leppla SH, Moayeri M (2010) Anthrax lethal toxin activates the inflammasome in sensitive rat macrophages. Biochem Biophys Res Commun 398: 785–789. 20638366 10.1016/j.bbrc.2010.07.039 PMC2925535

[6] Levinsohn JL, Newman ZL, Hellmich KA, Fattah R, Getz MA, et al (2012) Anthrax lethal factor cleavage of Nlrp1 is required for activation of the inflammasome. PLoS Pathog 8: e1002638. 22479187 10.1371/journal.ppat.1002638 PMC3315489

[7] Vitale G, Bernardi L, Napolitani G, Mock M, Montecucco C (2000) Susceptibility of mitogen-activated protein kinase kinase family members to proteolysis by anthrax lethal factor. Biochem J 352 Pt 3: 739–745. PMC1221512 11104681

[8] Turk BE, Huang LL, Piro ET, Cantley LC (2001) Determination of protease cleavage site motifs using mixture-based oriented peptide libraries. Nat Biotechnol 19: 661–667. 11433279 10.1038/90273

[9] Chopra AP, Boone SA, Liang X, Duesbery NS (2003) Anthrax lethal factor proteolysis and inactivation of MAP-kinase-kinase. J Biol Chem 278: 9402–9406. 12522135 10.1074/jbc.M211262200

[10] Frew B, Joag VR, Mogridge J (2012) Proteolytic processing of Nlrp1b is required for inflammasome activity. PLoS Pathog 8: e1002659. 22536155 10.1371/journal.ppat.1002659 PMC3334886

[11] D’Osualdo A, Weichenberger CX, Wagner RN, Godzik A, Wooley J, et al (2011) CARD8 and NLRP1 undergo autoproteolytic processing through a ZU5-like domain. PLoS ONE 6: e27396. 22087307 10.1371/journal.pone.0027396 PMC3210808

[12] Park S, Leppla SH (2000) Optimized production and purification of *Bacillus anthracis* lethal factor. Protein Expr Purif 18: 293–302. 10733882 10.1006/prep.2000.1208

[13] Wickliffe KE, Leppla SH, Moayeri M (2008) Anthrax lethal toxin-induced inflammasome formation and caspase-1 activation are late events dependent on ion fluxes and the proteasome. Cell Microbiol 10: 332–343. 17850338 10.1111/j.1462-5822.2007.01044.x PMC2515708

