# Supplementary figures and images for: Anthrax Lethal Factor Cleaves Mouse Nlrp1b in Both Toxin-Sensitive and Toxin-Resistant Macrophages
Source: PLoS One. 2012 Nov 12;7(11):e49741. doi: 10.1371/journal.pone.0049741 (PMC3495862; doi:10.1371/journal.pone.0049741)

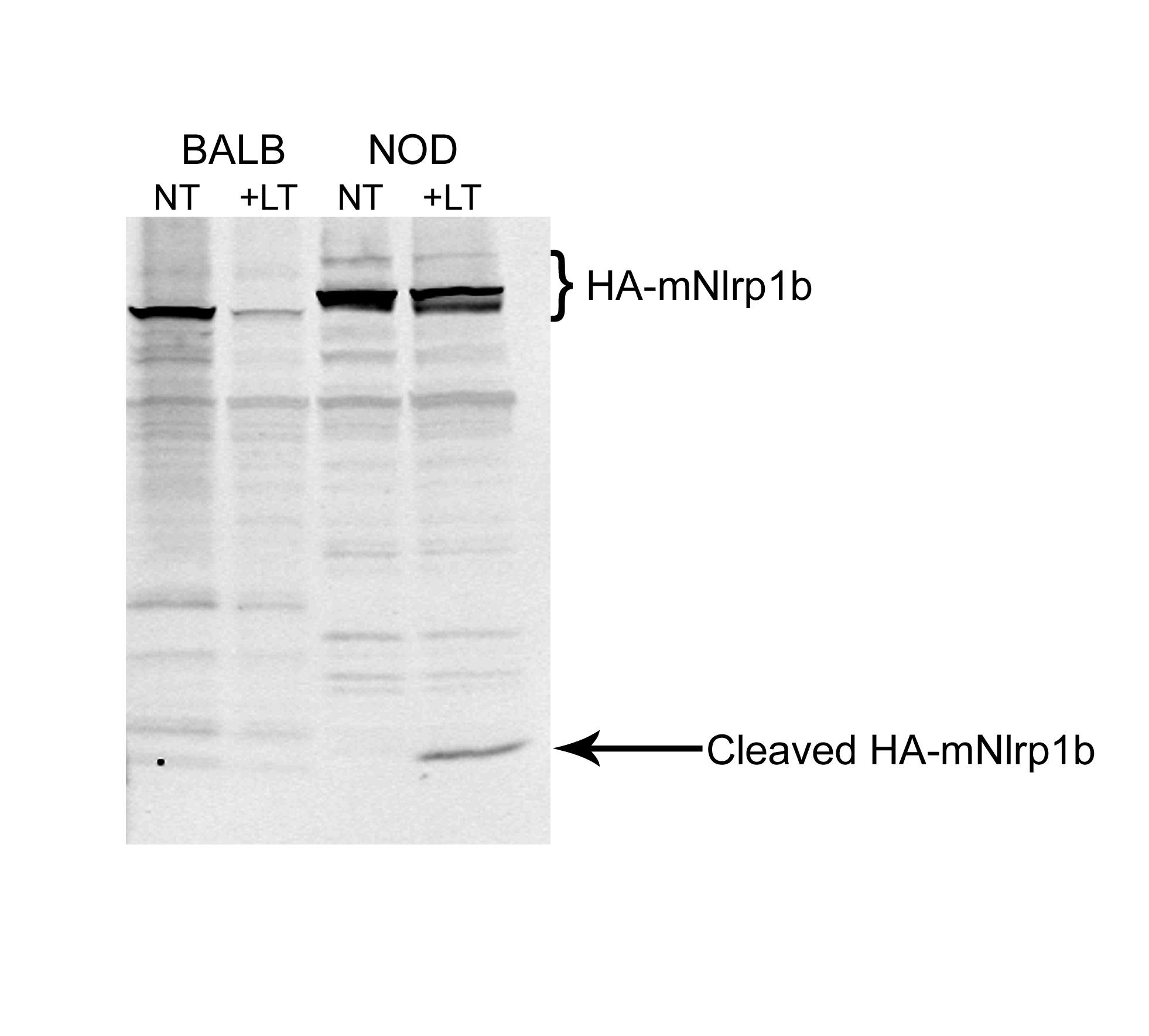

Supplement: Figure S1 — Canonical cleavage of full length mouse Nlrp1b proteins by LT. HT1080 cells expressing HA-tagged mouse Nlrp1b (BALB or NOD) proteins were first treated with LF+PA (1 µg/ml, each) for 3 h. IP (anti-HA pulldown) was then performed on lysates followed by anti-HA Western blotting. (TIF) [file pone.0049741.s001.tif]
